# Supplementary material for: egal-1 and microtubules promote regeneration polarity in planarians
Source: Development. 2025 Oct 16;152(20):dev204668. doi: 10.1242/dev.204668 (PMC12579938; doi:10.1242/dev.204668)
Supplement: Supplementary information [file develop-152-204668-s1.pdf]

A.

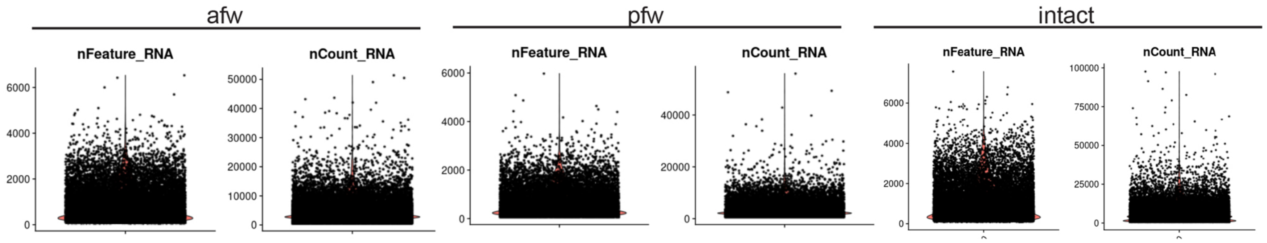

	number of cells	mean UMIs per cell	mean genes per cell
**afw**	8,486	5,828	1,143
**pfw**	7,466	5,098	904
**0 hpa**	6,602	7,768	1,268

B.

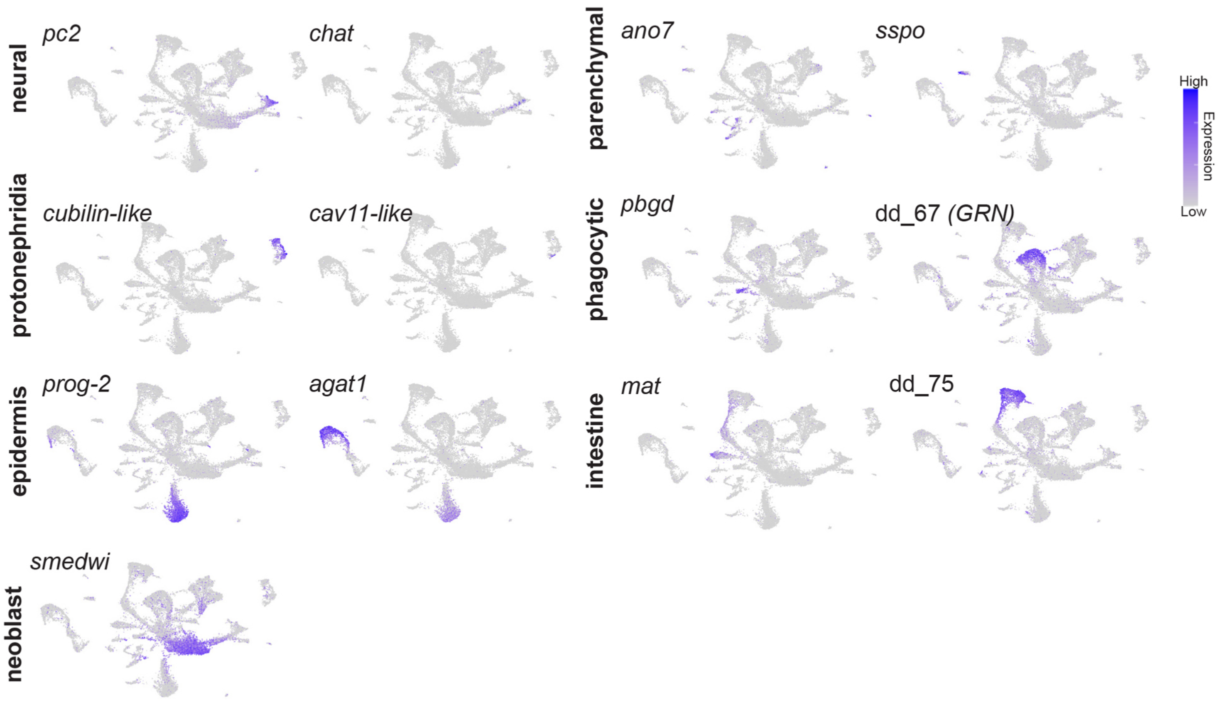

C.

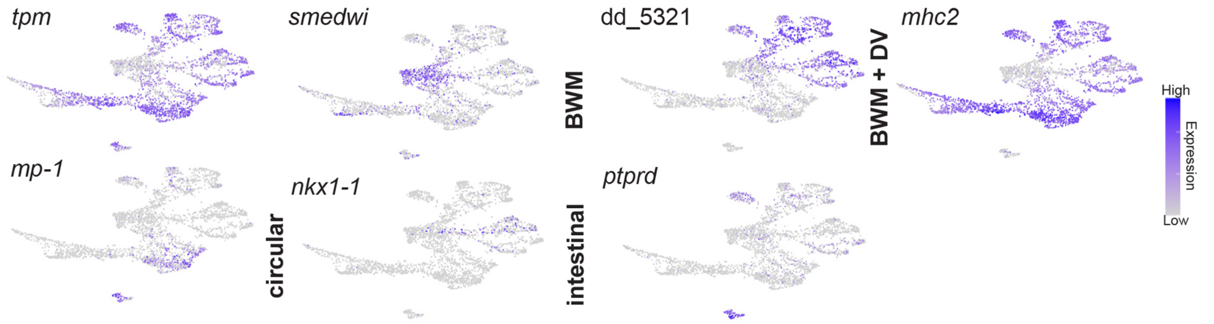

Supplementary Figure Legends

Fig. S1. Annotation of tissue clusters using tissue-specific markers

A. Violin plots show number of UMIs and genes per cell in each condition (anterior-facing wounds at 18 hpa, posterior-facing wounds at 18 hpa, and 0 hpa control) with the total number of genes detected per cell (nFeature) and mean UMIs per cell (nCount). Summary statistics are provided in the accompanying table.
B. UMAP plot of all cells showing the expression of tissue-specific markers.
C. UMAP plot of muscle cells shows expression of specific muscle fiber markers: dd_5321, body wall muscle (BWM); *mhc2,* BWM and dorsal-ventral (DV) fibers; *mp-1,* DV fibers; *nkx1-1*, circular fibers; dd_12771 *(PTPRD*), intestinal muscle.

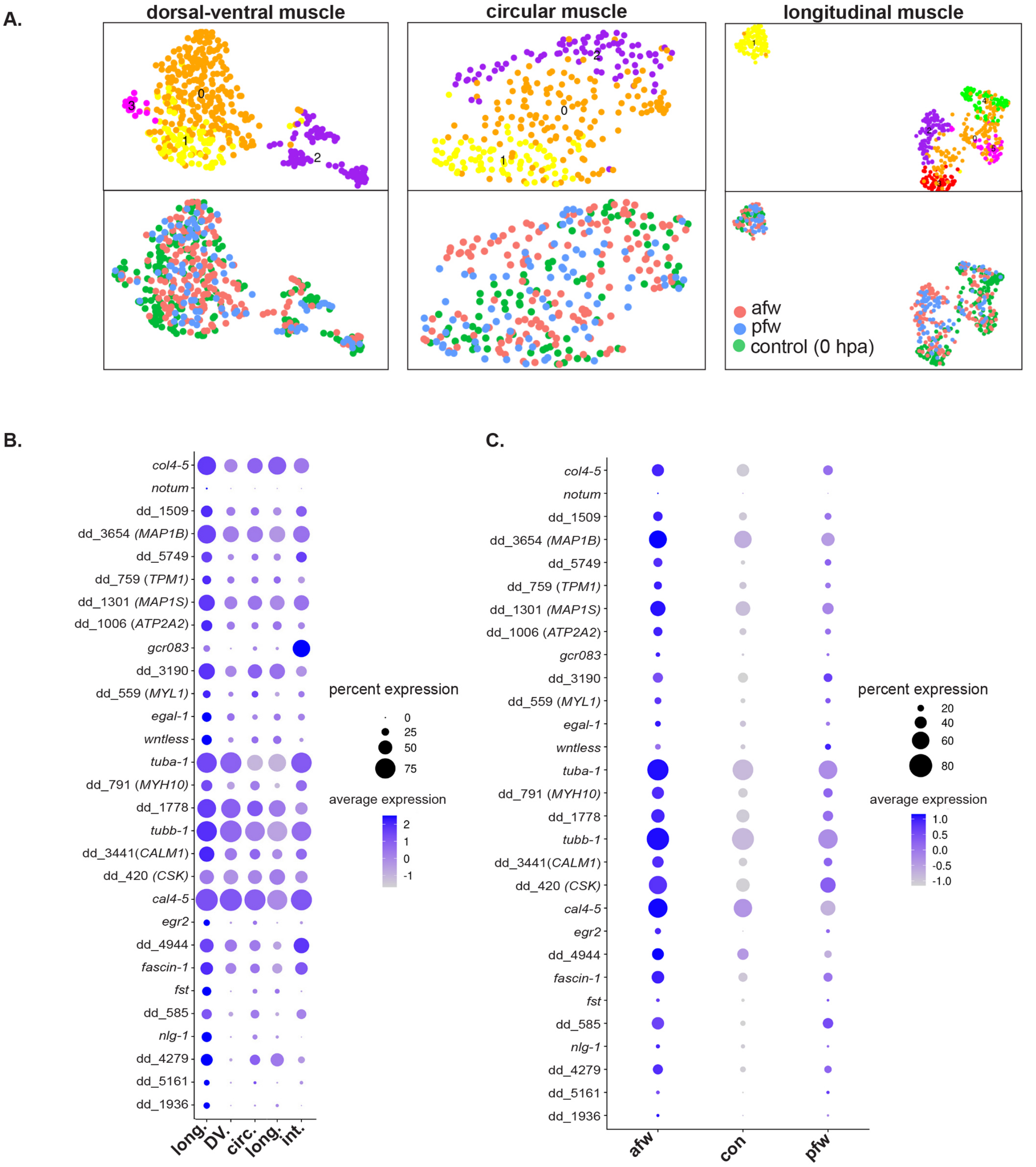

Fig. S2. Wounded muscle subclustering and expression of wound-induced genes

A. UMAP plot of subclustered muscle types reveals no distinct clusters lacking intact control in circular or DV muscle fibers.

B. Dot plot of expression of the top 30 genes enriched in the "wounded longitudinal muscle" cluster in different muscle types and

C. Expression of these genes in different conditions (afw, pfw, or control) shows higher levels of expression in longitudinal muscle at anterior- and posterior-facing wounds.

Development • Supplementary information

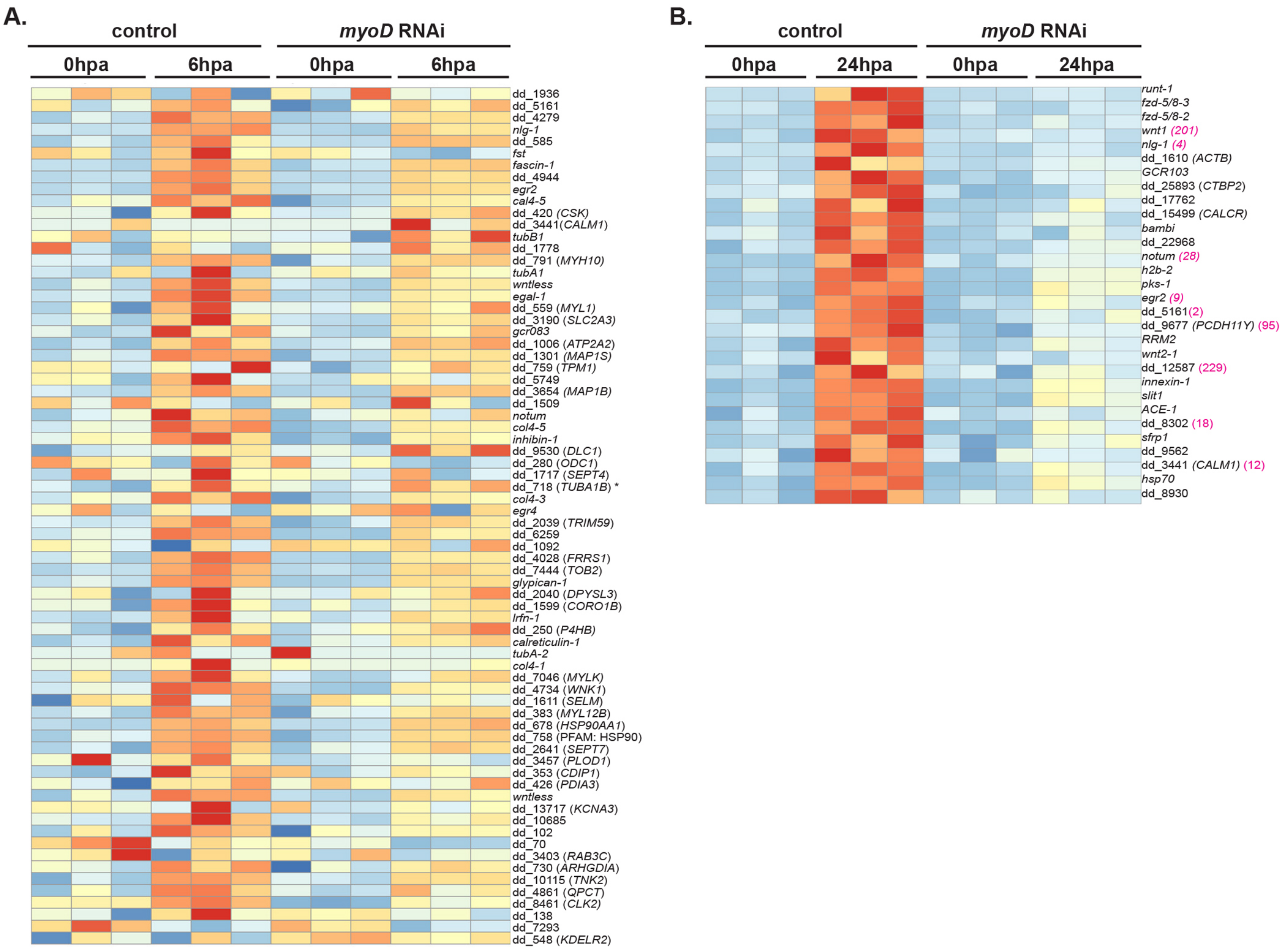

Fig. S3. **Bulk RNA-seq data heatmap comparing expression differences between control and *myoD* RNAi animals**.

A. and B. RNA-seq data heatmap from Scimone et.al (2017) dataset. Each column is a biological replicate. Data are Z-score normalized. (A) Expression of candidate wound-induced muscle genes from scRNA-seq data. (B) Heatmap of genes upregulated at 24 hpa compared to 0 hpa control animals. Top 20 genes at lower levels in injured *myoD* RNAi compared to controls. Numbers indicate ranking of enrichment of genes from the wound-induced longitudinal muscle subcluster.

Development • Supplementary information

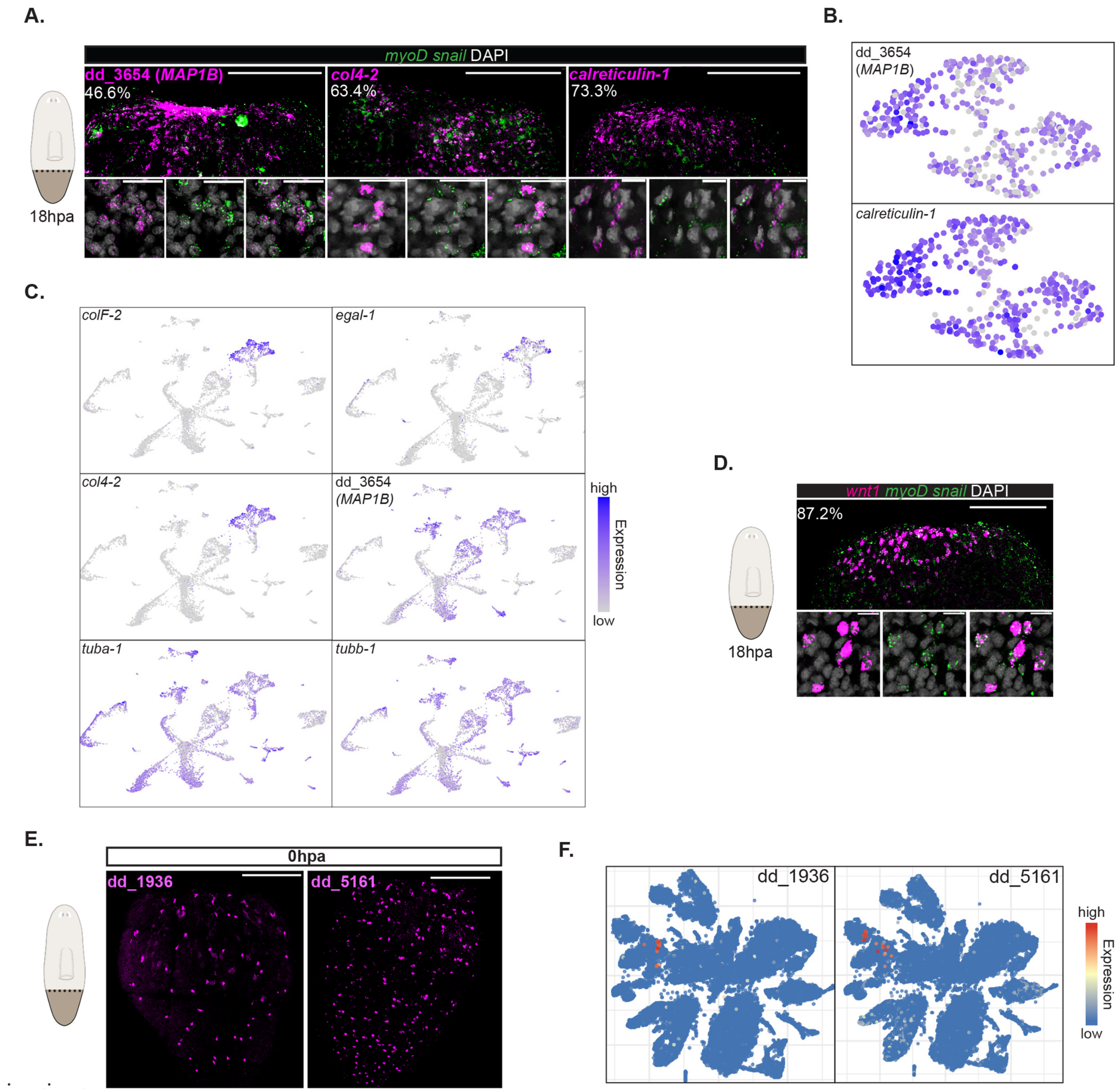

Fig. S4. Homeostatic and wound-induced gene expression by FISH and scRNA-seq.

A. Co-expression of pooled *myoD* and *snail* probes with muscle wound-induced genes showing wound-induced expression in longitudinal muscle at 18 hpa. (A, D) Co-expression counts were taken from the wound region, averaged from 3 animals in one experiment. Percentage indicates the fraction of cells expressing the wound-induced gene that co-localize with longitudinal muscle markers. Scale bars, 200 µm (top); 20 µm (bottom).

Development • Supplementary information

B. UMAP plot of selected genes indicate broad expression in the longitudinal muscle subclusters.
C. UMAP of control animals (0 hpa) showing *egal-1* and *col4-2* expression in *colF-2*+ muscle cell cluster and broad expression of dd_3654 (*MAP1B*)*, tuba-1, tubb-1* throughout different tissues.
D. Co-expression of pooled *myoD* and *snail* probes with *wnt1.*
E. dd_1936 and dd_5161 expression at 0 hpa in tail fragments. Scale bar, 20 μm.
D. UMAP plots depict expression of dd_1936 and dd_5161 in homeostatic animals from Fincher et. al (2018) data.

Development • Supplementary information

A.

	anterior regeneration						posterior regeneration					
	0hpa	1hpa	4hpa	12hpa	16hpa	24hpa	0hpa	1hpa	4hpa	12hpa	16hpa	24hpa
lrfn-1	-0.98	-0.91	1.88	1.48	-0.10	-0.34	-1.05	-1.16	1.84	0.87	-0.89	0.25
dd_1778	-1.13	-1.28	-0.43	1.23	1.12	1.07	-1.39	-0.98	-0.42	0.94	1.33	1.10
col4-2	-0.83	-0.24	-2.16	0.22	1.11	0.76	-0.64	-1.48	-0.91	1.11	1.20	1.25
calreticulin-1	-0.80	-0.57	-1.23	1.55	1.65	0.29	-0.29	-0.14	-1.08	0.94	2.02	0.46
dd_3441(*CALM1*)	-1.27	-1.14	-1.38	0.68	0.81	0.43	-0.91	-1.12	-0.68	1.39	1.60	0.64
dd_3654 (*MAP1B*)	-1.18	1.09	-0.25	1.47	0.72	-0.61	-0.81	0.86	-1.03	1.31	1.25	-0.39
dd_4028 (*FRSS1*)	-0.99	-1.96	-0.22	1.02	1.15	0.77	-1.25	-1.60	-0.15	1.14	1.09	1.12
dd_4279	-1.76	-1.22	0.18	1.44	0.85	0.44	-1.96	-1.04	-0.32	0.98	1.09	0.28
dd_4944	-0.79	-0.42	1.80	1.36	0.63	-0.66	-0.70	-0.35	1.92	1.19	0.59	-0.54
tuba-2	-0.82	-1.30	-1.05	-0.32	-0.05	-0.05	-1.23	-1.61	-0.81	0.03	0.61	0.29
dd_585	-1.46	-1.52	-0.54	0.85	0.90	1.10	-1.41	-1.50	-0.25	0.87	1.07	1.14
dd_7046	-1.31	-1.56	-1.06	0.89	0.69	0.77	-1.61	-1.31	-0.31	0.72	1.25	0.41
egal-1	-1.28	-1.16	0.97	1.32	0.99	0.57	-1.07	-0.99	1.40	1.36	0.95	-0.08
dd_8461 (*CLK2*)	-0.94	-1.28	-0.21	1.55	0.93	0.82	-1.06	-1.22	-0.30	1.67	0.86	0.71
egr2	-1.58	-0.70	1.07	1.22	0.88	0.70	-1.64	-0.61	1.18	1.03	0.81	0.75
fascin-1	-1.28	-1.52	0.32	1.41	1.17	0.39	-1.07	-1.24	0.61	1.52	1.22	0.27

B.

	anterior regeneration				
	0hpa	4hpa	12hpa	16hpa	24hpa
lrfn-1	-1.73	0.62	1.07	0.90	0.77
nlg-1	-2.35	-0.12	0.50	0.55	0.69
dd_1778	-1.09	-0.30	1.01	1.13	1.09
col4-2	-2.14	-0.68	0.56	0.48	0.67
calreticulin-1	-1.40	-1.02	1.51	0.91	0.81
notum	-1.84	-0.77	0.95	1.15	0.62
wnt1	-2.10	-0.61	0.66	1.19	0.60
dd_3441(*CALM1*)	-1.76	-1.08	0.39	0.21	0.94
dd_3654 (*MAP1B*)	-1.61	0.26	1.28	1.34	-0.42
dd_4028 (*FRSS1*)	-2.08	-0.32	0.92	1.06	0.71
dd_4279	-2.10	-0.27	0.86	0.89	0.74
dd_4944	-1.18	1.33	1.35	0.79	-0.53
tuba-2	-1.23	-0.89	-0.50	-0.45	-0.30
dd_585	-1.80	-0.65	0.86	0.96	1.11
dd_7046	-1.86	-1.15	0.42	0.52	1.06
inhibin-1	-1.49	-0.11	1.04	0.87	1.00
egal-1	-1.26	1.06	0.89	0.91	0.68
dd_8461 (*CLK2*)	-0.99	0.10	0.94	1.14	1.11
egr2	-1.81	1.04	0.92	0.83	0.45
wntless	-1.53	0.40	0.88	1.10	1.03
fst	-1.67	-1.05	0.95	1.17	0.79
fascin-1	-0.98	0.72	1.38	1.06	0.35

Fig. S5. Time-course of wound-induced gene expression

A. Heatmap of bulk RNA sequencing data from Wurtzel et.al (2015) and (B.) Liu et al (2013) shows early expression of *egal-1*, dd_4944, and *lrfn-1* after injury. Numbers are representative of log2 RPKM values.

Development • Supplementary information

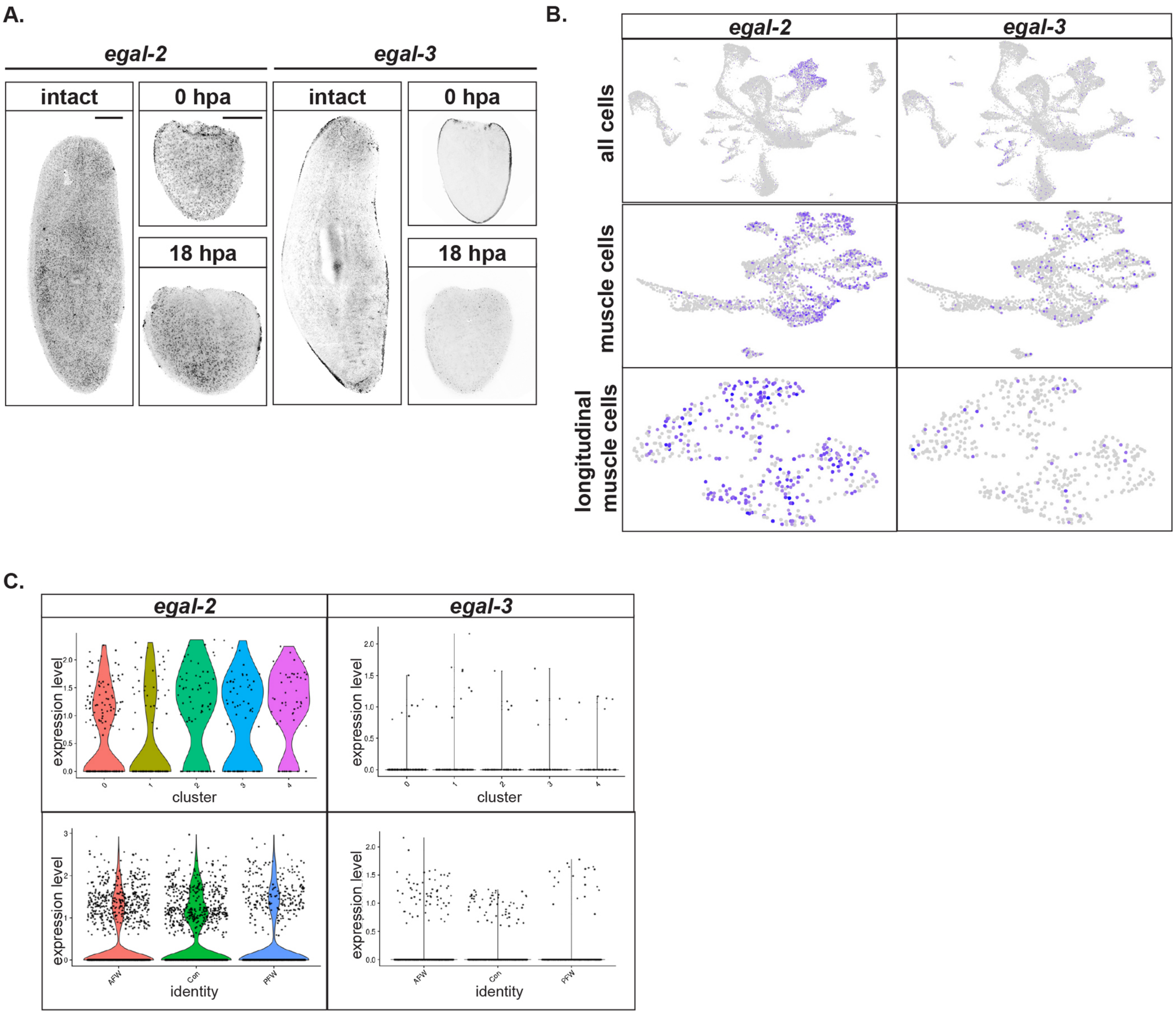

Fig. S6. *egal-2* and *egal-3* expression patterns

A. FISH showing *egal-2* uniform expression and *egal-3* sparse expression throughout the AP axis of intact animals. Neither show elevated expression in 18 hpa tail fragments by FISH. Scale bar, 200 μm.

B. UMAP plots show expression of *egal-2* in the muscle cell cluster and *egal-3* expression in the parenchymal cell clusters.

C. Violin plots showing expression levels of *egal-2* and *egal-3* in longitudinal muscle subclusters (top) and in all muscle cells grouped by tissue identity (bottom; anterior-facing wound [afw], posterior-facing wound [pfw], or control [con]). Cluster 1 (top) corresponds to the wound-induced muscle subcluster. Neither *egal-2* nor *egal-3* exhibit elevated expression in the wound-induced cluster or in wounding-associated tissue (afw, pfw).

Development • Supplementary information

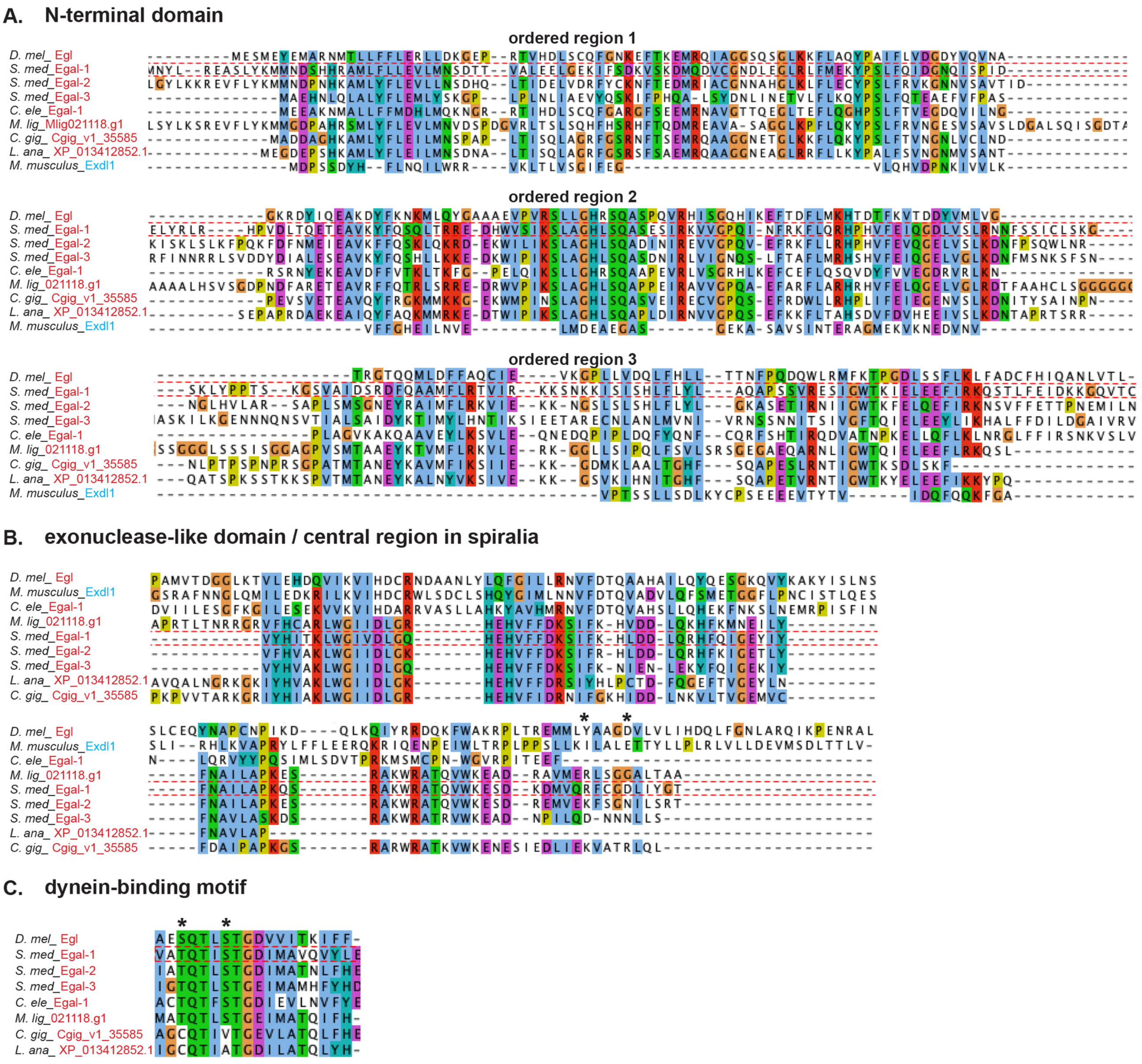

Fig. S7. Protein sequence alignment of Egalitarian-related proteins across species

A. Amino acid sequence alignment of N-terminal domain, (B) exonuclease-like domain/ central region and (C) dynein-binding motif of Egalitarian-related proteins across species. Colored by ClustalW colors. Red dotted box is around EGAL-1 of *Schmidtea mediterranea*. Asterisks represent (B) catalytic sites of exonuclease-like domain and (C) amino acids required for dynein binding in *D. melanogaster* Egl.

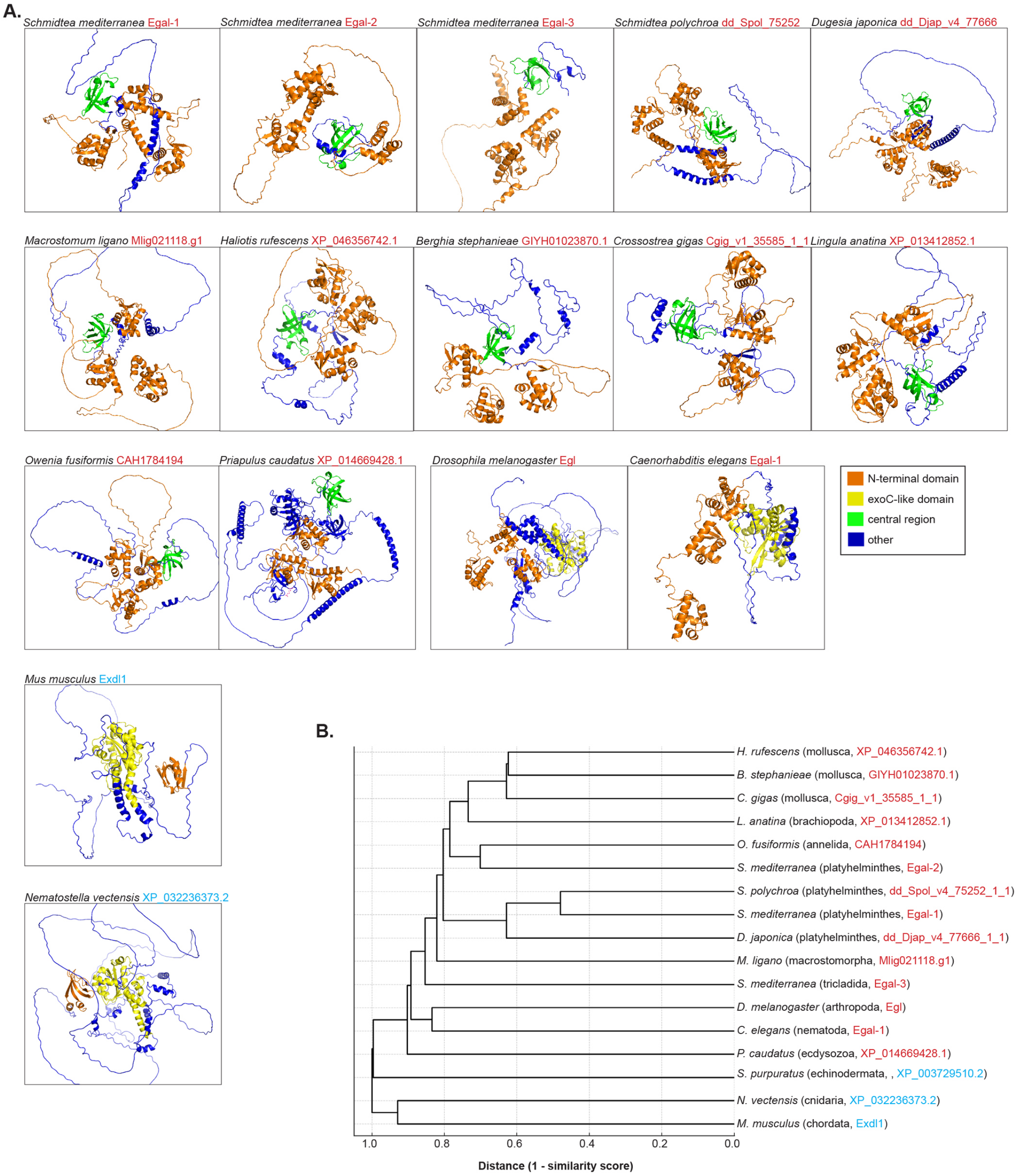

Fig. S8. Structural relationship of Egalitarian-related proteins across species.

A. AlphaFold2-predicted structures of candidate Egalitarian homologs across species show similar folding of three distinct structured regions within the N-terminal domain.

B. Structure-based dendrogram of protein relationships generated from Foldseek pairwise comparisons using the easy-search function to obtain structural similarity scores (bits). Dendrogram was generated using a distance metric of 1 – bits/max bits, with clustering performed using hierarchical agglomerative linkage. Tree topology reflects relative structural similarity among proteins.

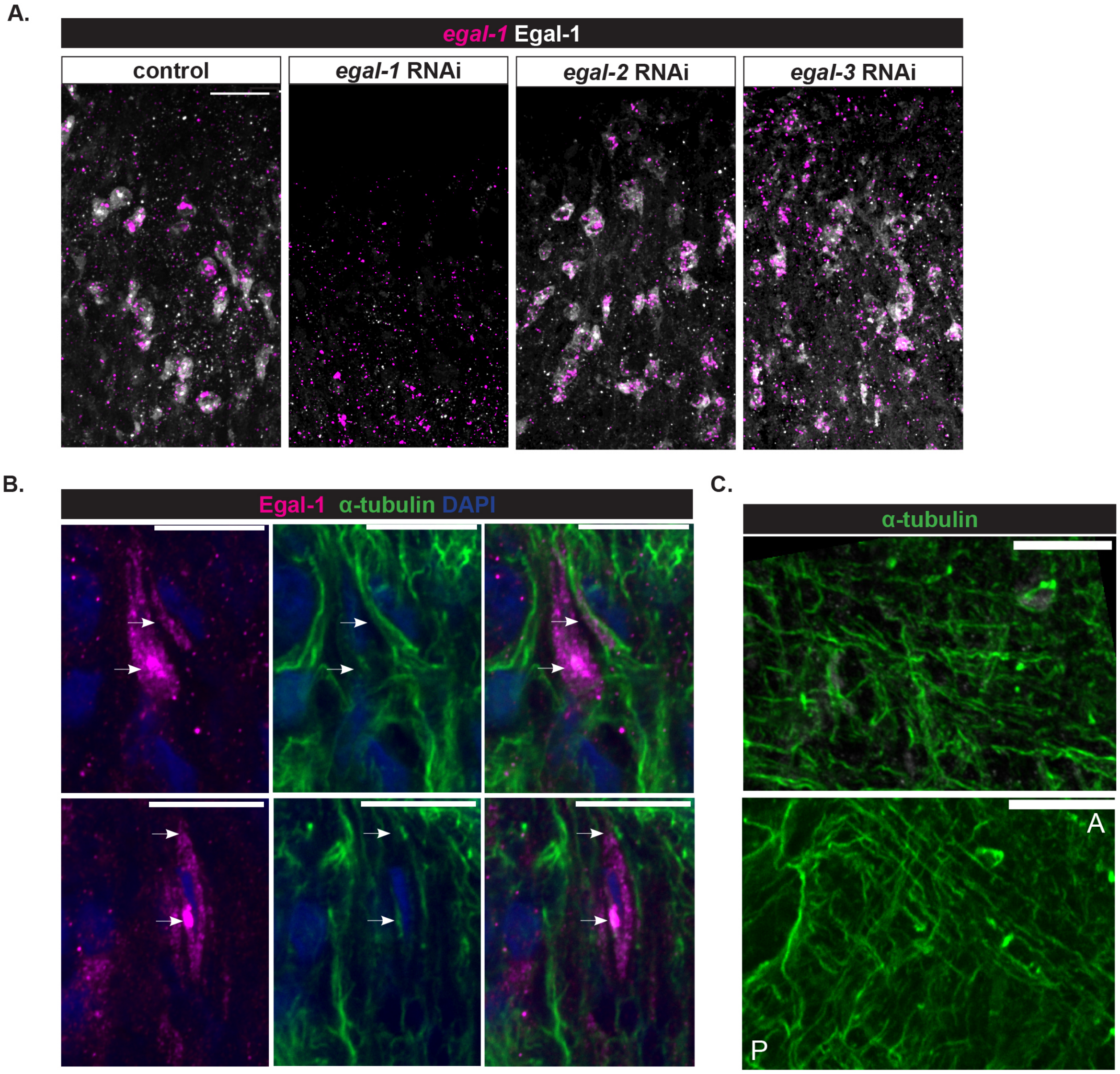

Fig. S9. Egal-1 antibody specificity and co-localization with α- tubulin

A. Co-expression of Egal-1 protein and *egal-1* transcript in *egal-2* and *egal-3* RNAi animals after 18 days of dsRNA feedings. Loss of Egal-1 protein and *egal-1* transcript expression in *egal-1* RNAi animals confirms Egal-1 antibody specificity. Wound region of 18 hpa tail fragments (subepidermal layer). Scale bar, 20 μm.

B. Immunofluorescence showing co-localization of Egal-1 with α-tubulin, a microtubule marker. Single z-stack image with arrows pointing to examples of co-localization at Egal-1^{+} perinuclear foci and within fibers.

C. α-tubulin network in the dorsal subepidermal layer of 18 hpa tail fragment shown by immunofluorescence. A (anterior); P(posterior). (B and C) Scale bar, 10 μm.

Development • Supplementary information

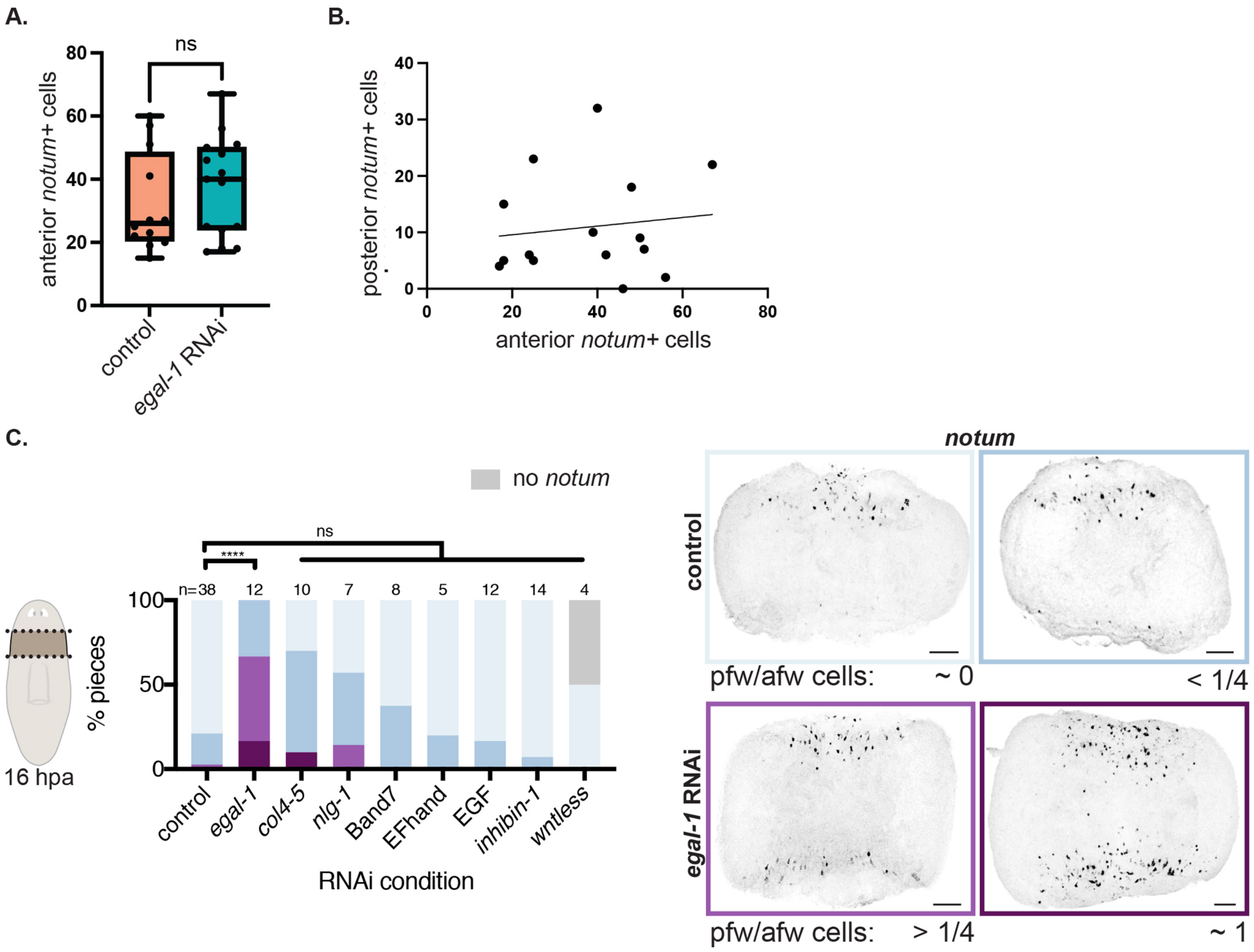

Fig S10. *notum* expression in anterior-facing wounds is not statistically different in *egal-1* RNAi animals and specificity of the *egal-1* RNAi phenotype.

A. The number of *notum*$^+$ cells at anterior-facing wounds in *egal-1* RNAi animals and control animals in Fig. 5A is not statistically different. (unpaired t-test, p-value= 0.37)

B. No correlation was observed between the number of *notum*$^+$ cells in posterior versus anterior-facing wounds in *egal-1* RNAi animals (R^2 = 0.017)

C. *egal-1* RNAi leads to ectopic *notum* expression at posterior-facing wounds at a significantly higher rate than other wound-induced genes (*inhibin-1, wntless, nlg-1*) and genes upregulated in longitudinal muscles at 18 hpa compared to 0 hpa (*col4-5*). The border colors correspond to posterior:anterior *notum*+ cell ratio. Light blue, pfw/afw = 0; blue, pfw/afw <1/4; purple, pfw/afw >1/4; dark purple, pfw/afw= 1.

Development • Supplementary information

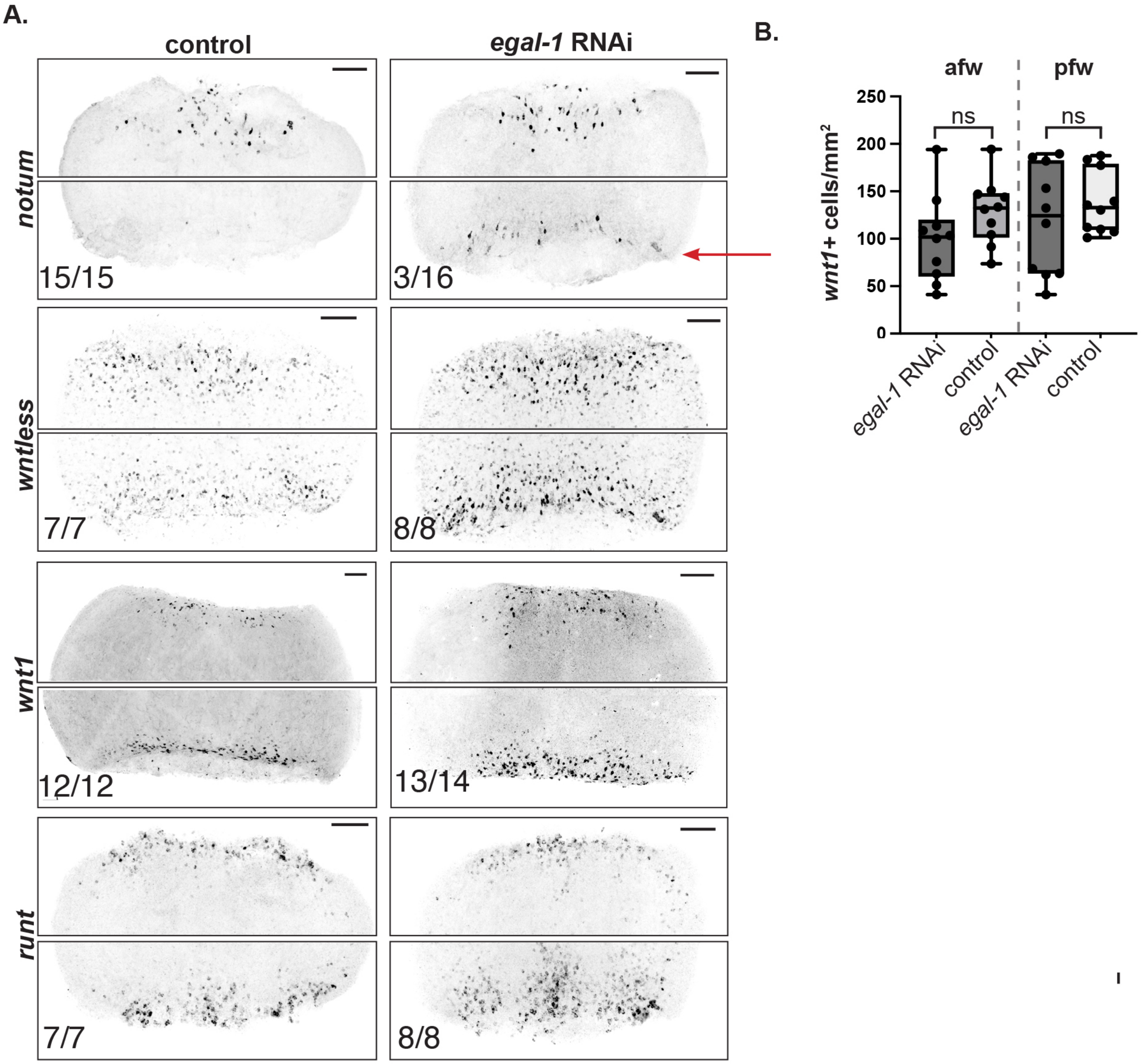

Fig S11. Wound-induced gene expression in *egal-1* RNAi animals

A. No detectable difference in expression of wound-induced genes (*wntless, wnt1, runt*) other than *notum* in *egal-1* RNAi animals. Animals were cut 4 days post final feeding and had 31 days of feeding, consisting of 8 feeds total. Scale bar, 100 μm.

B. Quantification of wound-induced *wnt1* at anterior-facing and posterior-facing wounds of pre-pharyngeal fragments normalized to counting area. (p-value pfw: 0.37; afw: 0.12)

Development • Supplementary information

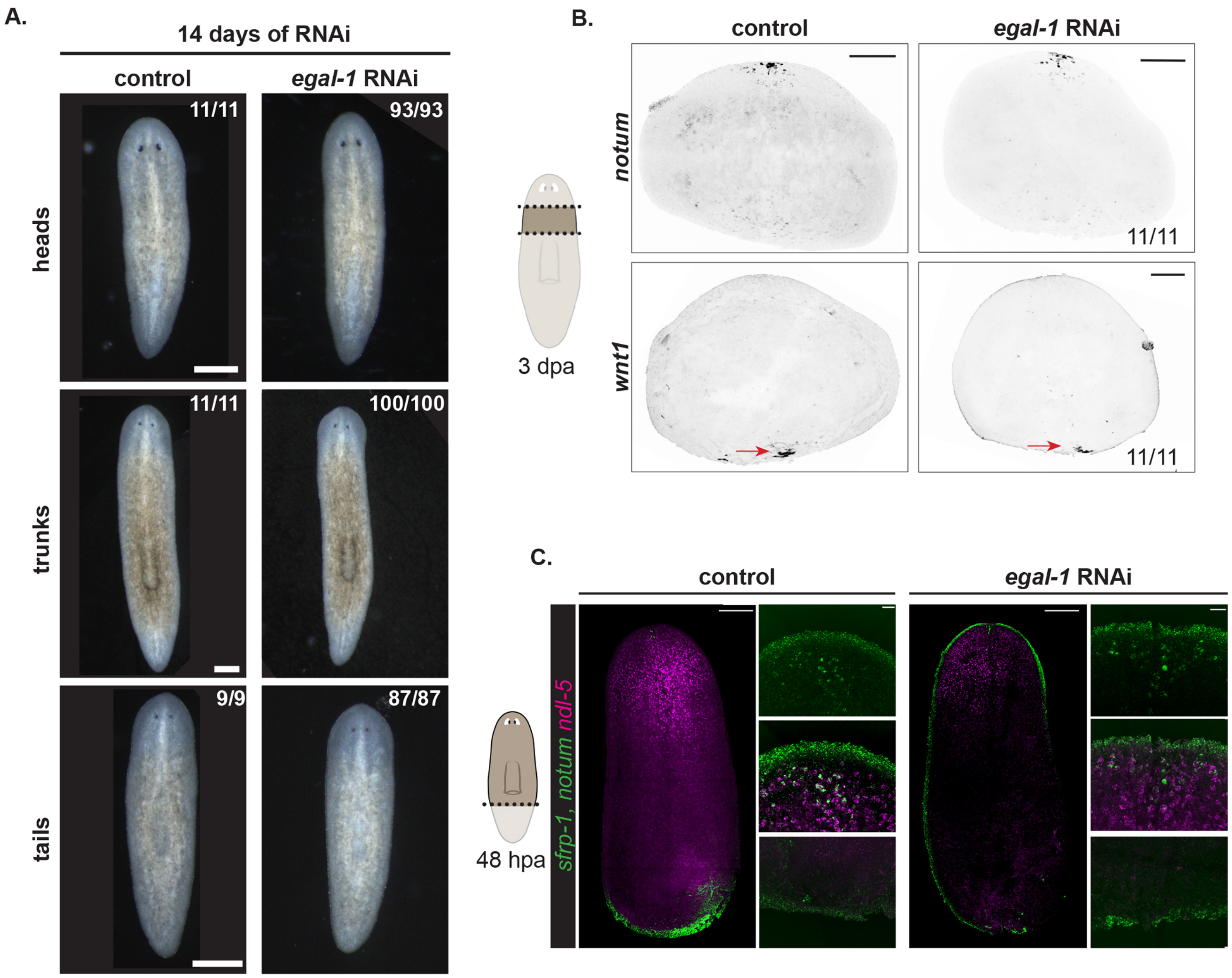

Fig S12. *egal-1* RNAi animals regenerating animals show no major PCG defects

A. Regeneration of head, trunk, and tail fragments following *egal-1* RNAi shows no detectable difference compared to control animals.

B. Anterior pole marker, *notum*, and posterior pole marker, *wnt1*, are expressed at the anterior and posterior end, respectively, of regenerating animals at 3 dpa in *egal-1* RNAi animals. Scale bar, 200 μm.

C. *notum*$^{+}$ anterior pole and anterior positional control genes, *sfrp-1* (green) and *ndl-5* (magenta) in *egal-1* RNAi showed no expression at the posterior wound of animals. Background signal observed around the periphery of the animal but does fluorescence signal was not around the cytoplasm of a single DAPI+ nucleus. Inset shows a comparable region of an independent animal. Scale bar, 200 μm (left); 20 μm (right).

Development • Supplementary information

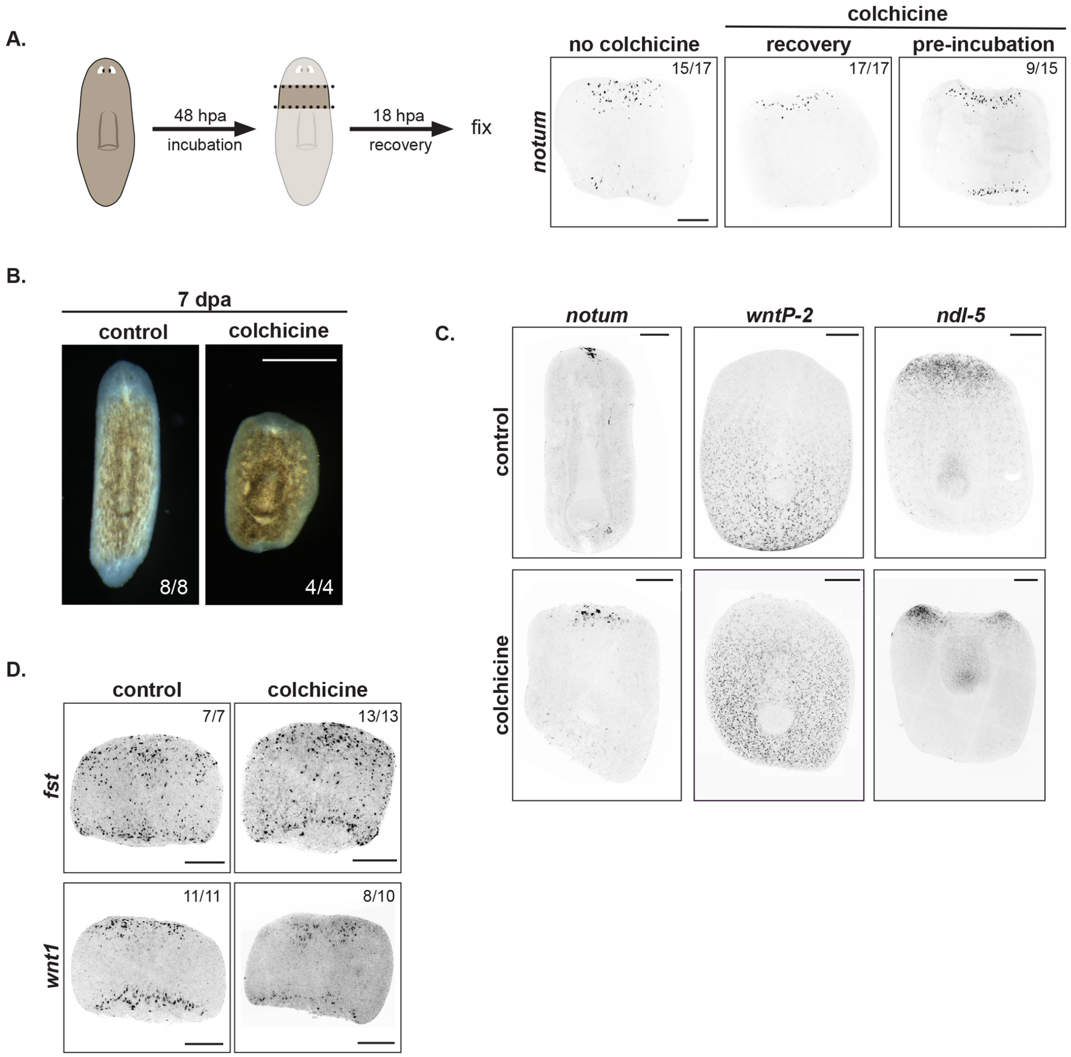

Fig S13. Characterization of colchicine-treated animals

A. Left: Depiction of experimental workflow. Right: *notum* expression at 18 hpa in animals treated with colchicine before amputation or during recovery only. Treatment with colchicine led to ectopic *notum* expression when treated before amputation but showed no defects when treated only during the recovery period.

B. Colchicine-treated animals have regeneration defects and improper blastema formation.

C. Dispersed *notum*$^+$ anterior pole in colchicine-treated animals compared to control. Anterior PCG, *ndl-5*, and posterior PCG, *wntP-2,* display proper AP patterning but *ndl-5* is expressed with some disruption in the middle of the wound site of colchicine-treated animals. 3 dpa regenerating trunk fragments.

D. Colchicine-treated animals show normal expression of the wound-induced genes *fst* and *wnt1*.

Scale bars, 200 μm.(A-D)

Development • Supplementary information

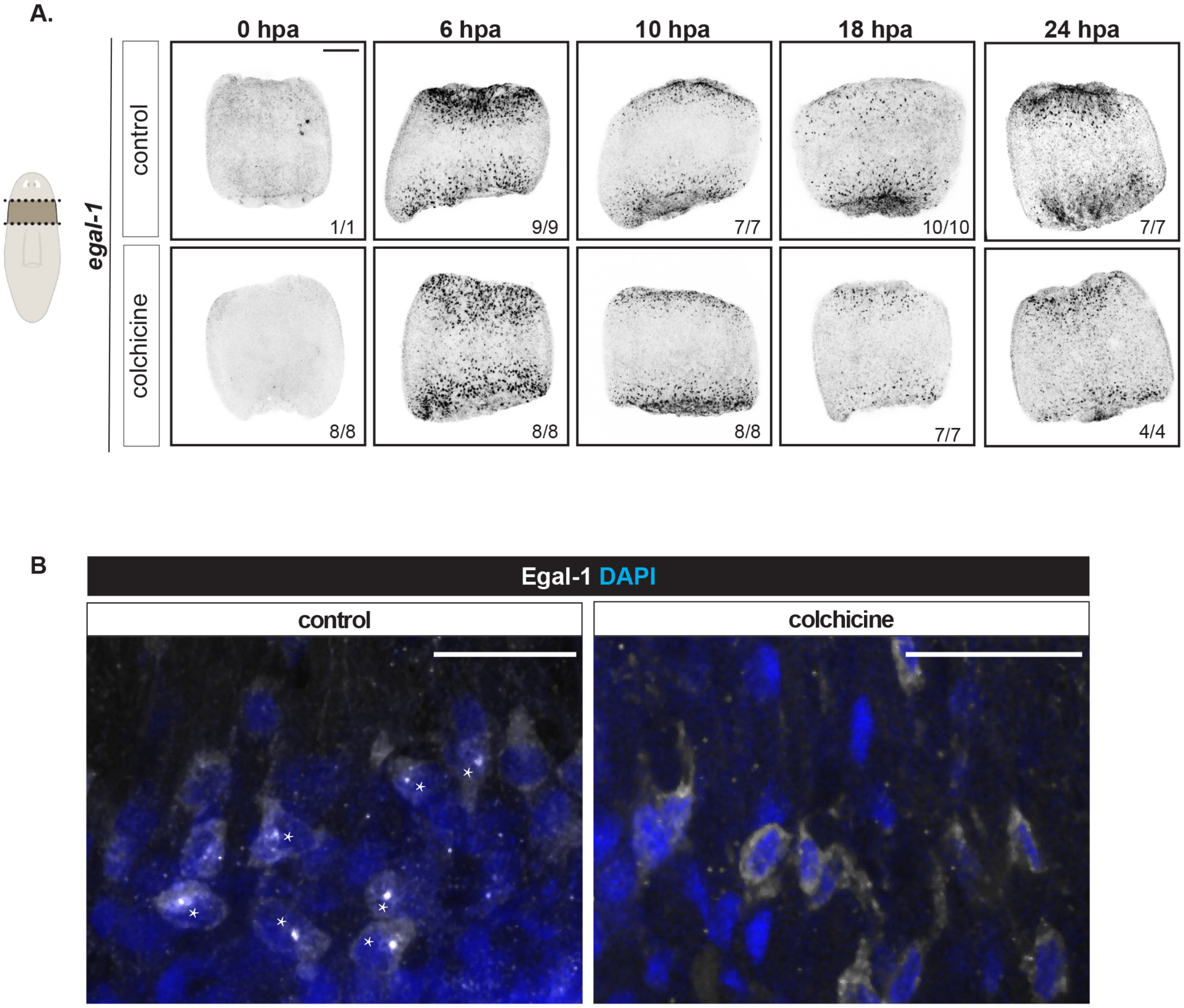

Fig S14. Colchicine treatment disrupts Egal-1 protein localization without altering transcript levels

A. Time course of *egal-1* expression in colchicine-treated animals shows comparable *egal-1* expression levels in control and treated animals. Scale bar, 200 µm.

B. Egal-1 perinuclear foci are disrupted with colchicine treatment. Asterisks indicate Egal-1$^+$ foci in control animals. Anterior-facing wound of tail fragments at 18 hpa. Scale bar, 20 µm.

Development • Supplementary information

Table S1. Markers for “wound-induced” longitudinal muscle cluster arranged by decreasing avg_log_2 fold change values

Available for download at
https://journals.biologists.com/dev/article-lookup/doi/10.1242/dev.204668#supplementary-data

Table S2. Bulk RNA-seq dataset from Scimone et al. (2017) showing genes downregulated in *myoD* RNAi animals at 6 hpa and 24 hpa after filtering for genes upregulated in control animals at 6 hpa and 24 hpa relative to 0 hpa.

Available for download at
https://journals.biologists.com/dev/article-lookup/doi/10.1242/dev.204668#supplementary-data

Table S3. Protein sequences used for homology analysis of Egalitarian-like-1

Available for download at
https://journals.biologists.com/dev/article-lookup/doi/10.1242/dev.204668#supplementary-data
